# Physician Choice Making and Characteristics Associated With Using Physician-Rating Websites: Cross-Sectional Study

**DOI:** 10.2196/jmir.2702

**Published:** 2013-08-28

**Authors:** Martin Emmert, Florian Meier, Frank Pisch, Uwe Sander

**Affiliations:** ^1^Institute of Management (IFM)School of Business and EconomicsFriedrich-Alexander-University Erlangen-NurembergNurembergGermany; ^2^University of Applied Sciences and ArtsHannoverGermany

**Keywords:** physician-rating website, public reporting, patient satisfaction, physician choice making

## Abstract

**Background:**

Over the past decade, physician-rating websites have been gaining attention in scientific literature and in the media. However, little knowledge is available about the awareness and the impact of using such sites on health care professionals. It also remains unclear what key predictors are associated with the knowledge and the use of physician-rating websites.

**Objective:**

To estimate the current level of awareness and use of physician-rating websites in Germany and to determine their impact on physician choice making and the key predictors which are associated with the knowledge and the use of physician-rating websites.

**Methods:**

This study was designed as a cross-sectional survey. An online panel was consulted in January 2013. A questionnaire was developed containing 28 questions; a pretest was carried out to assess the comprehension of the questionnaire. Several sociodemographic (eg, age, gender, health insurance status, Internet use) and 2 health-related independent variables (ie, health status and health care utilization) were included. Data were analyzed using descriptive statistics, chi-square tests, and *t* tests. Binary multivariate logistic regression models were performed for elaborating the characteristics of physician-rating website users. Results from the logistic regression are presented for both the observed and weighted sample.

**Results:**

In total, 1505 respondents (mean age 43.73 years, SD 14.39; 857/1505, 57.25% female) completed our survey. Of all respondents, 32.09% (483/1505) heard of physician-rating websites and 25.32% (381/1505) already had used a website when searching for a physician. Furthermore, 11.03% (166/1505) had already posted a rating on a physician-rating website. Approximately 65.35% (249/381) consulted a particular physician based on the ratings shown on the websites; in contrast, 52.23% (199/381) had not consulted a particular physician because of the publicly reported ratings. Significantly higher likelihoods for being aware of the websites could be demonstrated for female participants (*P*<.001), those who were widowed (*P*=.01), covered by statutory health insurance (*P*=.02), and with higher health care utilization (*P*<.001). Health care utilization was significantly associated with all dependent variables in our multivariate logistic regression models (*P*<.001). Furthermore, significantly higher scores could be shown for health insurance status in the unweighted and Internet use in the weighted models.

**Conclusions:**

Neither health policy makers nor physicians should underestimate the influence of physician-rating websites. They already play an important role in providing information to help patients decide on an appropriate physician. Assuming there will be a rising level of public awareness, the influence of their use will increase well into the future. Future studies should assess the impact of physician-rating websites under experimental conditions and investigate whether physician-rating websites have the potential to reflect the quality of care offered by health care providers.

## Introduction

Several studies have demonstrated variability in the quality of care across health care providers (eg, [1-5]). However, because of the limited amount of publicly reported quality information [6], patients are not likely to be aware of such differences [7,8]. To overcome this situation, public reporting instruments have been put in place within the past few years (eg, [9-16]). These instruments generally assess the quality of care by measuring adherence to clinical guidelines and provide some additional structural information [17].

Public reporting is supposed to increase the overall standard given by health care providers because it demonstrates which physicians use higher quality standards. This information steers patients to better performing providers; hence, motivating physicians to improve their overall quality outcomes [18,19]. In this context, previous systematic research has shown that public reporting has the potential to stimulate quality improvement outcomes at the hospital level. However, the effect on physicians or physician groups remains unaddressed [19]. Another review summarized the impact of 12 different public reporting instruments and included evidence from 21 studies, mostly from the United States. This study demonstrated that public reporting can be effective in directing patients when seeking a health care provider, especially for elective procedures [20]. Nevertheless, many authors state that patients have been slow to take advantage of comparative reports when making a health care provider choice (eg, [8]). Possible reasons for this are that patients are not aware of the information, do not understand it, do not believe it, or are not willing or able to use the information provided [21-23].

The newest trend in public reporting is physician-rating websites [24,25]. The primary objective of these relies on rating and discussing the physician’s standards by using user-generated data [25,26]. Such sites have been established in many countries worldwide, such as the United States, England, Germany, Canada, Australia, New Zealand, and the Netherlands [14,17,24,27-33]. Recent research in this field has focused on the number, distribution, and trend of evaluations on physician-rating websites [17,24,27-34]. It could be shown that approximately 1 in 6 physicians has been rated so far, and that approximately 90% of all ratings were positive [25]. Based on this information, it is assumed that the use of physician-rating websites will increase [24,25].

Thus far, no peer-reviewed research has focused on the influence of physician-rating websites when choosing a physician in the outpatient sector [28]. It still remains uncertain whether these websites have an influence on patients seeking and selecting a physician. Furthermore, it remains unclear what key predictors are associated with the knowledge and the use of physician-rating websites. In this context, this paper adds to the literature by investigating the influence of German physician-rating websites on patients choosing a physician in the outpatient sector and identifying the main predictors associated with the awareness and use of such sites.

## Methods

This study was designed as a cross-sectional survey. An online panel (Tomorrow Focus Media Opinion Pool) was consulted within Germany in January 2013. The online panel consisted of 3052 respondents who received €1 per finished survey. The panel consisted of online users who agreed to receive survey invitations about society or media-related topics once per month. They obtain bonus points that can be used for online shopping (eg, Amazon, Zalando, Douglas) or donations. The online survey was provided within the Tomorrow Focus Media Opinion Pool network. Several online channels were used to recruit participants for the survey. All panel members were invited via email and newsletter to participate (the invitation contained a link to enter the online survey). Additionally, online banner advertising was applied within the Tomorrow Focus Media network–related websites.

A questionnaire was developed containing 28 questions, addressing topics related to physician-rating websites (see Multimedia Appendix 1). The questionnaire was piloted by 50 individuals to ensure the comprehensibility of the wording and internal validity; final adjustments were made accordingly. The questionnaire first asked for the participants’ sources when seeking a physician. Participants were then asked 5 questions associated with the awareness and use of physician-rating websites, which served as our dependent yes/no variables and are described in the following (questions 1-3 included a list of the 9 leading German physician-rating websites for selection):

1. Have you ever heard of any of the following physician-rating websites? (awareness)
2. Have you ever searched for a physician on any of the following physician-rating websites? (searching)
3. Have you ever posted a rating on any of the following physician-rating websites? (rating)
4. Have you ever selected a particular physician based upon the publicly published results on any physician-rating website? (positive impact)
5. Have you ever not selected a particular physician based upon the publicly published results on any physician-rating website? (negative impact)

Further questions related to the importance of physician information provided on physician-rating websites, such as age, gender, medical devices, and number of patients treated by using a 5-point Likert scale from 1 (no importance at all) to 5 (very important). Following the specific physician-rating website questions, participants were asked a series of background sociodemographic questions (eg, age, gender, marital status, Internet use, and education). The survey ended with 2 health-related questions concerning the awareness of physician-rating websites and their usage. Health care utilization was measured in terms of the number of physician encounters within the past 6 months.

In addition to descriptive statistics, we used bivariate analyses (chi-square tests and *t* tests) to examine whether differences existed between those participants who stated that they were aware of or have used physician-rating websites and those who did not. Binary multivariate logistic regression was performed to identify the main predictors associated with the awareness and use of physician-rating websites (see previous description for the 5 dependent yes/no variables). Therefore, demographic data was dichotomized to contain group sizes to at least 20 participants in each subgroup [35]. For example, Internet use of the subgroups (ie, more than once a week, once a week, less than once a day, and once a month) was grouped as less than once a day. To ensure representativeness, the study sample was weighted for age, gender, and marital status according to the most recent data from the German Federal Statistical Office from 2011 [36]. Results from the logistic regression are presented for both the observed and the weighted sample.

Health status was measured by applying the World Health Organization (WHO) 5-item Well-being Index (WHO-5). The latter is comprised of 5 items, each rated on a 6-point Likert scale from 0 (not present) to 5 (constantly present); a raw score was calculated afterwards by summarizing the single scores. Higher scores reflect higher well-being status; conversely, poor well-being status is represented by a raw score below 13 or if the patient answered 0 to 1 on any of the 5 items [37].

All statistical analyses were conducted by using SPSS ver 21.0 (IBM Corp, Armonk, NY, USA). Observed differences were considered statistically significant if *P*<.05.

## Results

A total of 1505 respondents completed online interviews (response rate 49.28%) averaging 11.7 minutes. Regarding the study sample, 857/1505 respondents were female (57.25%), most were covered by statutory health insurance (SHI; 1173/1505, 80.67%), and the overall mean age was 43.73 (SD 14.39) years (see Table 1 for an overview of the study population). In all, 316 respondents (32.63%) had more than 5 encounters with a health care provider within the 6-month period before the survey.

The following are the results of our 5 main dependent variables (see Table 2 for an overview of the results). Approximately one-third (483/1505, 32.09%) of all respondents were aware of German physician-rating websites. Regarding the relative distribution of age, the highest awareness percentage was for the age range 31 to 40 years (37.01%). The lowest awareness was seen in the youngest age group, younger than 20 years (15.87%). However, differences between age groups were not proven to be statistically significant (*P*=.08). This is also true for differences regarding education, employment, Internet use, and health status. Statistically significant higher awareness levels were shown for female respondents (35.71% vs 27.50%, *P*<.001), those who were widowed (*P*=.012), covered by SHI (*P*=.02), and those with higher health care utilization (*P*<.001).

In all, 25.32% (381/1505) of the respondents reported to have actively searched for a physician using a German physician-rating website. Once more, statistically significant higher percentages could be shown for female respondents (*P*=.02), those who were widowed (*P*<.001), covered by SHI (*P*=.03), and those with higher health care utilization (*P*<.001). The highest active search percentage was calculated for the age ranges 31 to 40 years (29.18%) and 61 to 70 years (28.89%), respectively. With respect to employment, higher percentages were calculated for those who were unemployed compared to their employed counterparts (31.5%, *P*=.009).

Every ninth interviewee (166/1505, 11.03%) had already posted a rating on a physician-rating website. In other words, every third respondent who was aware of physician-rating websites (166/483, 34.37%) had already rated a physician. Differences with respect to marital status (*P*=.04), health insurance coverage (*P*=.04), and health care utilization (*P*<.001) were statistically significant. No meaningful differences were calculated for age, gender, Internet use, or other characteristics.

According to our results, physician-rating websites seem to have a meaningful influence on choosing a physician. For those respondents who had sought a physician online (381/1505, 25.32%), 327 respondents made their decision for a particular physician based on ratings shown on the websites. Considering this represents only one-quarter of respondents, not everyone has performed an online search using physician-rating websites. A physician search can also be performed on search engines, which are likely to transfer the seeker to the results presented on specific physician-rating websites. Consequently, it has to be distinguished between those respondents who use physician-rating websites to search for physicians and those who do not. Specifically, 249 respondents (249/381, 65.35%) claimed to have performed an online search on a physician-rating website and their decision was influenced on the provided ratings. Furthermore, those of younger age groups (≤40 years) have been influenced positively by the publicly reported data (*P*=.002); the highest percentages were reported for the age groups 21 to 30 years (33.01%) and 31 to 40 years (24.56%), respectively.

Conversely, physician-rating websites can have a meaningful negative influence on a patient’s choice. In our sample, 258 respondents (17.14%) did not consult a particular physician because of evaluation results on the websites. As mentioned previously, one has to distinguish between those respondents using physician-rating websites to search for physicians and those who do not. It was shown that 199 respondents (199/381, 52.23%) had performed an online search using a physician-rating website and made a subsequent decision against a particular physician. According to our results, younger study participants were significantly more influenced than their older counterparts were (*P*<.001). This was also true for female respondents (19.14% vs 14.69%, *P*=.02), those with higher education (*P*<.001), those employed (*P*=.04), and those with higher health care utilization (*P*=.003).

**Table 1:** Overview of study sample (N=1505).

Characteristics		Study sample
Age (years), mean (SD)		43.73 (14.39)
**Age range (years), n (%)**		
	≤20	63 (4.50)
	21-30	206 (14.70)
	31-40	281 (20.06)
	41-50	331 (23.63)
	51-60	306 (21.84)
	61-70	180 (12.85)
	>70	34 (2.43)
**Gender, n (%)**		
	Female	857 (57.25)
	Male	640 (42.75)
**Marital status, n (%)**		
	Married	713 (48.90)
	Single	560 (38.41)
	Divorced	149 (10.22)
	Widowed	39 (2.47)
**Education, n (%)**		
	High school	683 (46.62)
	Technical university entrance qualification	196 (13.38)
	Intermediate secondary school	345 (23.55)
	Polytechnic secondary school	71 (4.85)
	Secondary general school	148 (10.10)
	Without school qualification	2 (0.14)
	Others	20 (1.37)
**Employment, n (%)**		
	Self-employed	145 (9.85)
	Civil servants	68 (4.62)
	Employee	720 (48.91)
	Apprentices	24 (1.63)
	Unemployed	54 (3.67)
	Pensioners	202 (13.72)
	High school students	68 (4.62)
	Students (university/technical university)	92 (6.25)
	Others	99 (6.73)
**Health insurance, n (%)**		
	Statutory health insurance	1173 (80.67)
	Private health insurance	275 (18.91)
	No health insurance	6 (0.41)
**Health care utilization,** ^a^ **n (%)**		
	No treatment	138 (9.45)
	1	245 (16.77)
	2	312 (21.36)
	3	267 (18.28)
	4	183 (12.53)
	≥5	316 (21.63)
**Internet use, n (%)**		
	>once a day	1252 (83.19)
	once a day	178 (11.83)
	>once a week	68 (4.52)
	once a week	5 (0.33)
	>once a day	1 (0.07)
	once a month	1 (0.07)
**WHO-5 health status** ^b^		
	Overall, mean (SD)	14.53 (5.55)
	Poor (<13), n (%)	653 (44.30)
	Good (>), n (%)	821 (55.70)

^a^Number of encounters within the past 6 months.

^b^WHO-5 Well-being Index.

**Table 2:** Overview of survey results.

Variable		Awareness (n=483, 32.09%)		Searching (n=381, 25.32%)		Rating (n=166, 11.03%)		Positive impact (n=327, 21.73%)		Negative impact (n=258, 17.14%)	
		Mean (SD)/ n (%)	*P* ^a^	Mean (SD)/ n (%)	*P* ^a^	Mean (SD)/ n (%)	*P* ^a^	Mean (SD)/ n (%)	*P* ^a^	Mean (SD)/ n (%)	*P* ^a^
Age (years), mean (SD)		44.17 (14.06)	.44	44.61 (13.81)	.18	45.15 (14.37)	.19	41.57 (13.90)	.003	41.80 (13.86)	.02
**Age range (years), n (%)**											
	≤20	15.87	.08	4.76	.007	1.59	.14	22.22	.002	12.70	<.001
	21-30	33.50		27.18		11.65		33.01		29.61	
	31-40	37.01		29.18		12.81		24.56		16.73	
	41-50	30.51		24.77		9.37		20.24		16.31	
	51-60	32.03		24.51		11.44		19.28		14.71	
	61-70	32.78		28.89		14.44		13.89		12.22	
	>70	35.29		26.47		11.76		11.76		8.82	
**Gender, n (%)**											
	Female	35.7	<.001	27.8	.02	10.5	.40	24.2	.01	19.1	.02
	Male	27.5		22.3		11.9		18.8		14.7	
**Marital status, n (%)**											
	Married	31.4	.01	25.4	<.001	11.4	.04	20.2	.37	15.3	.25
	Single	30.7		22.0		8.8		22.5		18.6	
	Divorced	36.2		33.6		15.4		26.2		20.1	
	Widowed	55.6		52.8		19.4		25.0		22.2	
**Education, n (%)**											
	High School	35.1	.27	28.0	.17	11.3	.29	23.9	.31	20.9	<.001
	Technical university entrance qualification	28.1		22.4		12.2		20.4		15.8	
	Intermediate secondary school	32.5		27.8		11.3		22.3		17.1	
	Polytechnic secondary school	31.0		21.1		7.0		22.5		8.5	
	Secondary general school	29.7		19.6		12.8		16.2		9.5	
	Without school qualification	50.0		50.0		50.0		50.0		0.0	
	Others	15.0		15.0		0.0		10.0		0.0	
**Employment, n (%)**											
	Self-employed	35.2	.06	29.0	.009	9.0	.14	22.1	.02	19.3	.04
	Civil servants	33.8		26.5		14.7		25.0		20.6	
	Employee	32.4		26.7		11.4		24.3		19.6	
	Apprentices	33.3		29.2		12.5		37.5		29.2	
	Unemployed	42.6		31.5		11.1		25.9		16.7	
	Pensioners	31.2		27.2		14.9		15.3		11.9	
	High School Students	13.2		4.4		2.9		13.2		10.3	
	Students (university/technical university)	32.6		23.9		5.4		20.7		15.2	
	Others	34.3		20.2		10.1		14.1		10.1	
**Health insurance**											
	Statutory health insurance	34.4	.02	27.4	.03	12.0	.04	23.2	.07	17.7	.79
	Private health insurance	25.8		19.6		6.9		17.1		16.0	
	No health insurance	16.7		16.7		0.0		33.3		16.7	
**Health care utilization**											
	No treatment	20.3	<.001	13.8	<.001	3.6	<.001	10.1	<.001	8.7	.003
	1	24.5		19.2		5.7		16.7		15.1	
	2	34.0		26.9		12.2		20.5		14.4	
	3	30.3		24.3		9.4		22.8		19.1	
	4	34.4		27.9		14.2		25.1		19.7	
	5+	43.4		34.8		17.4		30.4		22.8	
**Internet use**											
	> once a day	33.2	.32	26.4	.33	11.4	.67	22.7	.33	17.9	.14
	once a day	25.3		18.5		7.9		15.2		10.7	
	> once a week	29.4		25.0		13.2		22.1		22.1	
	once a week	40.0		20.0		0.0		20.0		0.0	
	> once a day	0.00		0.0		0.0		0.0		0.0	
	once a month	0.00		0.0		0.0		0.0		0.0	
**WHO-5 health status**											
	Overall, mean (SD)	14.53 (5.59)	.97	14.43 (5.72)	.66	15.12 (6.24)	.15	14.55 (5.62)	.95	14.56 (5.36)	.94
	Poor (<13), n (%)	31.4	.58	25.4	.95	10.3	.31	21.6	.71	16.8	.73
	Good (>), n (%)	32.8		25.6		11.9		22.4		17.5	

^a^ *P* value was calculated using chi-square test or *t* test.

In connection with the demographic- and health-related variables, health care utilization was significantly associated with all dependent variables in our binary multivariate logistic regression models (see Tables 3 and 4). Additionally, gender, health insurance status, and health care utilization were all strongly associated with awareness of physician-rating websites. Awareness results were significantly higher in female (OR 0.75, 95% CI 0.57-0.98, *P*=.04), those insured by SHI (OR 0.63, 95% CI 0.42-0.94, *P*=.03), and those with a higher number of physician encounters (OR 4.16, 95% CI 2.34-7.38, *P*<.001). The awareness tended to be higher in widowed respondents, those with a higher education level, self-employed, frequently use the Internet, and those with a good health status. However, these differences were not statistically significant. It could further be shown that health insurance status and health care utilization were the only 2 independent variables which were proven to be strongly associated with the rating activity on physician-rating websites. Scores were significantly higher in participants insured by SHI (OR 0.48, 95% CI 0.25-0.92, *P*=.04) and those with a higher number of physician encounters (OR 7.47, 95% CI 2.21-25.27, *P*<.001). With respect to the last dependent variables of interest (ie, being positively or negatively influenced in choosing a physician by the results on physician-rating websites), only health care utilization could be shown to be strongly associated.

Tables 5 and 6 show the results of the weighted binary multivariate logistic regression models. After controlling for age, gender, and marital status (according to the German population in 2011 [36]), health care utilization and Internet use were shown to be significantly associated in all 5 models. Both education and health insurance status could further be shown to be strongly associated with searching for physicians on physician-rating websites (*P*<.05). As shown, a higher education level and being insured by SHI (OR 0.56, 95% CI 0.34-0.90, *P*<.05) indicate higher ratios. Furthermore, labor and health status were strongly associated with the rating activity on physician-rating websites. The same is true for marital status, which negatively influences the choice made when using the results on physician-rating websites. Only age and gender did not reach statistical significance in any of the weighted multivariate models.

**Table 3:** Independent factors associated with physician-rating website relevant issues (unweighted sample).

Variables		Awareness			Searching			Rating			Positive impact			Negative impact		
		OR	95% CI	*P*	OR	95% CI	*P*	OR	95% CI	*P*	OR	95% CI	*P*	OR	95% CI	*P*
Age				.80			.87			.99			.99			.99
**Gender**																
	Female	1.00			1.00			1.00			1.00			1.00		
	Male	0.75	0.57, 0.98	.04	0.81	0.61, 1.08	.13	1.35	0.91, 1.99	.22	0.84	0.62, 1.14	.22	0.79	0.57, 1.11	.13
**Marital status**				.36			.04			.53			.69			.17
	Married	1.00			1.00			1.00			1.00			1.00		
	Single	1.02	0.73, 1.41	.74	0.85	0.60, 1.21	.47	0.88	0.54, 1.44	.99	1.01	0.70, 1.45	.87	1.13	0.76, 1.68	.42
	Divorced	1.09	0.70, 1.68	.63	1.29	0.82, 2.01	.24	1.28	0.71, 2.30	.29	1.38	0.85, 2.22	.23	1.46	0.85, 2.51	.13
	Widowed	2.24	0.97, 5.17	.08	2.92	1.26, 6.75	.02	1.70	0.57, 5.03	.23	1.32	0.50, 3.48	.82	2.58	0.97, 6.89	.07
**Education**				.25			.16			.32			.76			.13
	Matura examination	1.00			1.00			1.00			1.00			1.00		
	Technical university entrance qualification	0.74	0.49, 1.10	.16	0.80	0.52, 1.22	.30	1.01	0.58, 1.78	.90	0.94	0.60, 1.46	.78	0.83	0.51, 1.34	.53
	Intermediate secondary school	0.83	0.59, 1.15	.26	0.90	0.64, 1.28	.57	0.86	0.53, 1.41	.52	0.93	0.64, 1.34	.83	0.79	0.53, 1.19	.25
	Polytechnic secondary school	0.76	0.41, 1.42	.42	0.55	0.27, 1.09	.10	0.28	0.08, 0.99	.045	1.14	0.58, 2.24	.57	0.33	0.12, 0.89	.03
	Secondary general school	0.76	0.47, 1.22	.25	0.58	0.34, 0.99	.04	1.15	0.61, 2.19	.92	0.70	0.39, 1.25	.30	0.44	0.22, 0.88	.03
	Others	0.21	0.05, 0.98	.045	0.33	0.07, 1.54	.15	0.28	0.03, 2.82	.22	0.57	0.14, 2.24	.31	0.00	0.00,	.99
**Labor**				.45			.58			.37			.25			.17
	Self-employed	1.00			1.00			1.00			1.00			1.00		
	Civil servants	0.90	0.43, 1.87	.78	0.83	0.38, 1.80	.62	2.76	0.89, 8.58	.15	1.08	0.48, 2.42	.94	1.13	0.47, 2.71	.94
	Employee	0.63	0.39, 1.01	.07	0.63	0.38, 1.04	.09	1.46	0.64, 3.36	.35	0.81	0.47, 1.38	.40	0.88	0.49, 1.57	.43
	Apprentices	0.78	0.36, 1.69	.60	0.77	0.34, 1.73	.55	0.60	0.16, 2.25	.46	0.64	0.28, 1.48	.30	0.57	0.23, 1.41	.13
	Unemployed	0.84	0.39, 1.83	.71	0.82	0.36, 1.85	.63	1.09	0.31, 3.90	.99	0.87	0.37, 2.08	.56	0.87	0.32, 2.33	.53
	Pensioners	0.55	0.30, 1.01	.06	0.64	0.34, 1.20	.17	1.90	0.73, 4.92	.21	0.52	0.26, 1.07	.06	0.49	0.22, 1.08	.05
	Others	0.71	0.37, 1.35	.43	0.47	0.23, 0.96	.047	1.46	0.50, 4.24	.37	0.44	0.20, 0.97	.05	0.41	0.17, 1.02	.07
**Health insurance**																
	Statutory health insurance	1.00			1.00			1.00			1.00			1.00		
	Private health insurance	0.63	0.42, 0.94	.03	0.61	0.40, 0.93	.04	0.48	0.25, 0.92	.04	0.70	0.44, 1.09	.14	0.81	0.50, 1.32	.41
**Health care utilization**				<.001			<.001			<.001			<.001			.003
	No treatment	1.00			1.00			1.00			1.00			1.00		
	1	1.80	0.99, 3.27	.05	1.71	0.89, 3.31	.10	2.26	0.62, 8.25	.21	1.87	0.91, 3.84	.07	1.95	0.92, 4.16	.08
	2	3.05	1.71, 5.44	<.001	2.88	1.53, 5.42	<.001	5.04	1.48, 17.18	.01	2.35	1.17, 4.70	.01	1.76	0.83, 3.72	.16
	3	2.61	1.44, 4.71	.001	2.48	1.30, 4.74	.005	3.61	1.03, 12.71	.04	2.49	1.23, 5.05	.008	2.57	1.22, 5.45	.02
	4	2.72	1.46, 5.06	.002	2.73	1.39, 5.37	.003	5.46	1.54, 19.42	.005	3.60	1.74, 7.46	<.001	2.77	1.27, 6.05	.008
	5+	4.16	2.34, 7.38	<.001	3.75	2.00, 7.00	<.001	7.47	2.21, 25.27	<.001	4.07	2.05, 8.05	<.001	3.67	1.78, 7.57	<.001
**Internet use**				.13			.06			.17			.06			.14
	> once a day	1.00			1.00			1.00			1.00			1.00		
	once a day	0.72	0.47, 1.10	.09	0.63	0.40, 1.01	.05	0.59	0.30, 1.18	.06	0.63	0.38, 1.05	.06	0.61	0.34, 1.10	.07
	< once a day	0.69	0.37, 1.30	.21	0.69	0.36, 1.35	.18	1.04	0.45, 2.38	.66	0.68	0.33, 1.39	.14	1.01	0.48, 2.14	.80
Health Status				.99			.99			.26			.72			.98

**Table 4:** Binary multivariate logistic regression analysis associated with physician-rating website relevant issues (unweighted sample).

Statistical results	Awareness	Searching	Rating	Positive impact	Negative impact
–2 Log-likelihood	1468.31	1332.10	778.44	1225.13	1040.82
Pseudo *R* ^*2*^ (Nagelkerke)	0.162	0.166	0.205	0.155	0.175
Constant	0.008	–20.176	–19.176	–20.011	–20.389
n	1279	1279	1279	1279	1279

**Table 5:** Independent factors associated with physician-rating website relevant topics (weighted sample).

Variables		Awareness			Searching			Rating			Positive impact			Negative impact		
		OR	95% CI	*P*	OR	95% CI	*P*	OR	95% CI	*P*	OR	95% CI	*P*	OR	95% CI	*P*
Age				.08			.47			.99			.60			.99
**Gender**																
	Female	1.00			1.00			1.00			1.00			1.00		
	Male	0.77	0.57, 1.03	.08	0.91	0.66, 1.26	.58	1.40	0.89, 2.19	.14	0.86	0.62, 1.21	.40	0.80	0.54, 1.17	.25
**Marital status**				.49			.56			.25			.90			.04
	Married	1.00			1.00			1.00			1.00			1.00		
	Single	1.03	0.70, 1.50	.21	0.85	0.53, 1.34	.48	1.15	0.59, 2.22	.68	0.90	0.55, 1.48	.69	1.07	0.62, 1.84	.81
	Divorced	1.02	0.63, 1.74	.96	1.05	0.58, 1.89	.89	1.56	0.71, 3.40	.27	1.00	0.52, 1.91	.99	1.13	0.54, 2.36	.75
	Widowed	1.49	0.65, 3.39	.34	1.69	0.73, 3.92	.22	2.62	0.94, 7.29	.07	1.35	0.53, 3.47	.53	4.18	1.59, 10.96	.004
**Education**				.07			.03			.57			.84			.32
	High School	1.00			1.00			1.00			1.00			1.00		
	Technical university entrance qualification	0.65	0.41, 1.03	.06	0.72	0.44, 1.18	.19	0.96	0.49, 1.87	.90	0.94	0.57, 1.57	.82	0.94	0.54, 1.64	.84
	Intermediate secondary school	0.81	0.55, 1.19	.28	0.90	0.60, 1.34	.59	0.99	0.56, 1.75	.97	1.00	0.64, 1.55	.99	0.88	0.54, 1.43	.60
	Polytechnic secondary school	0.68	0.34, 1.35	.27	0.44	0.21, 0.95	.04	0.39	0.12, 1.27	.19	1.28	0.59, 2.78	.53	0.25	0.07, 0.86	.03
	Secondary general school	0.76	0.45, 1.30	.32	0.45	0.24, 0.83	.01	0.87	0.41, 1.88	.73	0.84	0.43, 1.64	.61	0.62	0.29, 1.34	.22
	Others	0.10	0.02, 0.63	.01	0.21	0.03, 1.31	.10	0.29	0.03, 2.54	.27	0.43	0.10, 1.84	.25	0.00	0.00,	.99
**Labor**				.11			.50			.005			.26			.15
	Self-employed	1.00			1.00			1.00			1.00			1.00		
	Civil servants	0.91	0.39, 2.15	.83	1.28	0.51, 3.19	.60	4.84	1.33, 17.64	.02	1.57	0.60, 4.09	.36	1.77	0.61, 5.13	.29
	Employee	0.57	0.32, 1.00	.05	0.63	0.34, 1.15	.13	1.49	0.56, 3.99	.42	0.94	0.48, 1.83	.85	0.97	0.46, 2.03	.93
	Apprentices	0.64	0.29, 1.42	.27	0.66	0.28, 1.54	.34	0.43	0.11, 1.64	.216	0.91	0.37, 2.21	.83	0.65	0.24, 1.78	.41
	Unemployed	0.57	0.21, 1.55	.27	0.59	0.20, 1.74	.34	0.54	0.08, 3.61	.53	0.54	0.16, 1.77	.31	0.41	0.10, 1.71	.22
	Pensioners	0.35	0.18, 0.70	.003	0.59	0.28, 1.23	.16	1.22	0.41, 3.58	.72	0.46	0.20, 1.10	.08	0.49	0.19, 1.26	.14
	Others	0.73	0.34, 1.55	.41	0.58	0.25, 1.35	.20	2.38	0.69, 8.19	.17	0.60	0.23, 1.54	.29	0.47	0.15, 1.43	.18
**Health insurance**																
	Statutory health insurance	1.00			1.00			1.00			1.00			1.00		
	Private health insurance	0.77	0.50, 1.19	.24	0.56	0.34, 0.90	.02	0.52	0.25, 1.06	.07	0.82	0.50, 1.36	.45	0.79	0.46, 1.38	.41
**Health care utilization**				<.001			<.001			.002			<.001			<.001
	No treatment	1.00			1.00			1.00			1.00			1.00		
	1	2.32	1.17, 4.57	.02	2.02	0.96, 4.28	.07	1.82	0.51, 6.52	.36	1.88	0.82, 4.27	.13	2.05	0.83, 5.08	.12
	2	3.62	1.86, 7.03	<.001	3.26	1.57, 6.77	.002	3.57	1.06, 12.00	.04	2.62	1.18, 5.81	.02	1.62	0.65, 4.01	.30
	3	3.94	1.99, 7.79	<.001	3.63	1.73, 7.66	<.001	2.91	0.83, 10.25	.10	4.43	1.99, 9.86	<.001	4.25	1.75, 10.31	<.001
	4	3.23	1.60, 6.53	<.001	3.46	1.60, 7.49	.002	5.09	1.45, 17.86	.01	4.75	2.08, 10.86	<.001	3.90	1.56, 9.74	.004
	5+	4.85	2.49, 9.43	<.001	4.10	1.98, 8.48	<.001	6.66	2.00, 22.20	.002	5.85	2.64, 12.82	<.001	5.81	2.46, 13.76	<.001
**Internet use**				.03			.02			.03			.010			.01
	> once a day	1.00			1.00			1.00			1.00			1.00		
	once a day	0.53	0.32, 0.88	.01	0.46	0.26, 0.81	.007	0.28	0.11, 0.70	.007	0.45	0.25, 0.84	.011	0.37	0.18, 0.78	.009
	< once a day	0.61	0.29, 1.28	.19	0.57	0.26, 1.26	.17	1.02	0.37, 2.83	.97	0.41	0.17, 1.00	.05	0.44	0.15, 1.26	.13
Health status				.40			.14			.02			.06			.37

**Table 6:** Binary multivariate logistic regression analysis associated with physician-rating website relevant issues (weighted sample).

Statistical results	Awareness	Searching	Rating	Positive impact	Negative impact
–2 Log-Likelihood	1304.15	1160.57	703.95	1068.71	881.74
Pseudo *R* ^*2*^ (Nagelkerke)	0.276	0.268	0.294	0.259	0.278
Constant	–0.763	–20.163	–17.551	–19.926	–21.055
n	1279	1279	1279	1279	1279

## Discussion

### Principal Findings

Research in the field of public reporting has primarily focused on the effects of traditional instruments, which provide quality information about health care providers as related to clinical measures. However, little knowledge is available about the awareness and influence of physician-rating websites on a patient’s choice. It remains unclear which key predictors are associated with the knowledge and the use of such sites. In this context, this study investigates the influence of physician-rating websites when choosing a physician and it identifies the main key predictors that are associated with the knowledge and the use of physician-rating websites by conducting a cross-sectional online survey.

In our study, approximately one-third (483/1505, 32.09%) of all respondents were aware of the existence of German physician-rating websites. This demonstrates that physician-rating websites are likely to have achieved a significant amount of publicity at least when it comes to the online population so far. Numbers from the United States indicate lower levels of awareness for such websites, although the data are older. In 2008, the Update on Consumers’ Views of Patient Safety and Quality Information telephone-based survey (N=1517 respondents) showed that only 6% of Americans had heard of Hospital Compare [38], a consumer-oriented website that provides information on how well hospitals provide recommended care to their patients [39]. Another telephone survey was conducted in 2007 (N=1007 Californian adults) that showed that less than one-quarter of respondents (22% in 2007 vs 14% in 2004) had seen physician quality ratings; however, those numbers are rising [40].

In our study, one-quarter of respondents (381/1505, 25.32%) had actively searched for a physician on a German physician-rating website. Compared with other previous German surveys, this indicates an increasing amount of users on such websites. In 2011, the German Society for Consumer Research (Gesellschaft für Konsumforschung) showed a slightly lower percentage (22.6%) [41]. In 2011, another representative telephone survey of 2048 German citizens showed only 10% of respondents had searched for physicians by using German physician-rating websites (7% in 2010) [42,43]. The differences might, to a certain degree, be because of the study population (online panel vs telephone survey). In 2010, in the United States, a telephone-based survey among 3001 adults was conducted and it found that 16% of current Internet users and 19% of current online health seekers had consulted online rankings or reviews of doctors or of other providers. The same was true of another survey conducted in December 2008, which reported that 24% of respondents had used an online ranking or review when choosing a physician [44]. In general, 12% of adults have consulted online rankings or reviews of doctors or of other providers [45]. In 2011, 5% of US consumers had reported using a blog in the past year to learn about others’ health care experiences (in the report, the term “blog” was not defined; thus, it remains uncertain whether these blogs are equal to physician-rating websites) [46].

Concerning our sample, 11.03% (166/1505) of respondents had posted a rating on a German physician-rating website. With reference to respondents who were exclusively aware of physician-rating websites, every third respondent had already rated a physician. The numbers observed here are higher than those from other studies. The representative telephone survey of 2048 German citizens mentioned previously showed that only 2% of respondents had posted a rating for physicians on German physician-rating websites in 2011 [42]; 1 year before (in 2010), the number was only 1% [43]. A telephone-based US survey among 3001 adults in 2010 found that 4% of current Internet users (n=2065) and 6% of current online health seekers had posted an online review of a doctor. This was consistent with another study conducted in December 2008 that reported 5% [44]. Two additional studies found that 3% of adults had posted a review online about a doctor [45,46]. Therefore, only a minority have posted a rating on a physician-rating website. Rating numbers from other sectors confirm this observation (music: 5%, real estate: 4%, and cell phone: 3%) [47].

There are some surveys that investigate the impact of publicly available quality information on consumer behavior. According to our study, 65.35% (249/381) of those having performed an online search by means of a physician-rating website made their decision based on the ratings presented. This gives leverage to the statement that physicians should not underestimate the impact of such sites. Because patient awareness of such sites is likely to grow, it can be inferred that patients will be increasingly influenced by the information presented on physician-rating websites. In a US survey from 2007, it was shown that 14% of Internet users read online reviews before purchasing medical services. Of those, 76% specified that these online reviews had a significant influence on their decision [48]. However, another US study from 2008 found a much lower impact. The California telephone survey conducted in 2007 showed that only 2% of those surveyed had made a change based on information posted on a rating site (1% in 2004) [40]. Numbers from other sectors have also shown a lower impact on decision making (eg, music: 7%, cell phone: 10%) [47].

In our models, the most strongly associated variable for our physician-rating website measure was shown to be health care utilization. This is in-line with a large study conducted by Andreassen et al [49], who investigated factors that affect the health-related use of the Internet among 7 European countries. They also showed statistically significant higher odds ratios in the subsample of Internet users with higher health care utilization. Moreover, a statistically significant number of those insured by SHI were likely to be aware of such sites and use them more often when seeking and rating a physician online. Although we could not find any published evidence backing our finding, it seems probable that this is because of the fact that some large physician-rating websites are administered by SHI companies (eg, the Arztnavigator is run by the largest German SHI, Allgemeine Ortskrankenkasse). They have been promoting their website through various media channels, such as television, newspaper, radio, Internet, and membership magazines. This may have led to higher scores for those insured by an SHI company.

Higher odds ratios were calculated for female respondents in 4 variables, although only differences with respect to the awareness of the sites were proven to be statistically significant. However, significant differences could not be shown in any of our weighted models. Higher health-related online activity levels for females in general have been shown by various other studies that confirm our finding [49-57]. One explanation for that finding might be that women are more interested in health-related Internet use than are men [48,58]. Furthermore, women are more likely to register strong positive beliefs regarding the benefits of online health searches [59]. Additionally, it seems likely that it is mostly females who take responsibility for the family’s health. In cases of illness, it is mostly females who seek medical aid for themselves, their husbands, or their children [58].

In almost every model, those who were widowed were more likely to be aware of, or take advantage of, physician-rating websites, although differences were statistically significant on only 2 accounts: participants who actively searched for physicians in the unweighted models and participants who were negatively influenced in the weighted models. Because these participants had already lost a family member, it seems likely that some might have searched for health- and/or disease-related information online. Possibly, they came across physician-rating websites and were, therefore, more familiar with those websites. However, we did not find any evidence backing our assumption. In contrast, those widowed were likely to be older and possibly not familiar with online websites. Studies have shown that widowers, in general, have a lower use of eHealth [57]. Other studies have found that individuals who are married or who live with a partner are more likely to search for health information online [51,53]. We could not prove whether there were any statistically significant differences in our results regarding age in any of our models. In general, other studies (mostly telephone-based surveys) have shown that online health information seekers are relatively young (eg, [49-53,57,60,61]). However, there has not been sufficient amount of research conducted with a focus on obtaining an online sample.

No significant differences could be demonstrated with respect to education, employment, Internet use, or health status in our unweighted models. The latter is interesting because one could assume higher use of such sites with poorer health status. This assumption is backed by French and Italian evidence, which shows statistically significant higher eHealth use results for respondents with a poor perception of health or mental health as compared with those of moderate or excellent health perceptions [53,57]. However, several studies have been published showing similar results to ours. For example, Couper et al [50] demonstrated that those with better self-rated health had higher scores for health-related Internet use than those with lower health status (although not statistically significant). Andreassen et al [49] also showed an opposite impact of health status on health-related Internet use (ie, those who reported poorer health used the Internet less for health purposes). Neither Hüfken and colleagues [61] nor Dumitru and colleagues [62] could prove higher health-related Internet use for those with poorer health status. However, medical indicators of health, such as a current diagnosis of long-term illness or disability, indicate a higher level of health-related use of the Internet [49]. Regarding education, our results are in-line with other studies showing that people with higher education are more likely to use the Internet for health purposes (eg, [49,53,56,57,60]). Higher results for those with higher education levels could be demonstrated in almost all models, although differences could not be proven to be statistically significant. Finally, concerning the frequency of Internet use, no statistically significant differences could be observed in the unweighted models, but they could be found in almost all of the weighted models. Higher results for those respondents using the Internet more frequently could also be shown in other studies (eg, [53,56]).

In summary, this study demonstrated that physician-rating websites have become more common in the German online environment. Compared with previous investigations, the number of users seems to have increased. This is especially true of females with a higher education status, who are insured by SHI, and who utilize the health care sector at a higher rate. This group has demonstrated that it is aware of, and that it takes advantage of, such sites. The strongest predictor for physician-rating website use was shown to be health care utilization. Finally, it should be emphasized that physician-rating websites play an important role in choosing a physician and since their emergence in the public domain, they have influenced the decision-making process of patients. With a further increase in popularity of such sites, we predict that their influence will likely increase. Future studies are needed to investigate whether physician-rating websites have the potential to reflect the quality of care offered by health care providers.

### Limitations

There are some limitations that have to be taken into account when interpreting the results of this investigation. Firstly, this study was designed as a cross-sectional survey. Thus, we were able to identify association between exposure and outcomes. However, we could not infer cause and effect. Furthermore, the findings about the influence of physician-rating websites might differ from those studies applying an experimental design under real conditions. Therefore, we did not analyze empirical data regarding the influence in terms of numbers of encounters per quarter, the change with respect to the proportion of SHI to private health insurance patients per practice, etc. Next, we consulted an online panel for our study purposes. Obtaining an online sample instead of an offline sample meant that representation of a sample population as a whole (including online and offline samples) was not achievable. (According to the D21-Digital-Index-2013, approximately 23.5% of the German population are offline [63].) Even adjusting for differences in age, gender, education, etc, cannot compensate for the offline population. As a consequence, our findings may not be generalizable to the entire German population because the composition of the study population is predominantly middle-aged, female, and covered by private health insurance. Our study is also limited because of surveying an online panel. Those participants might be more familiar with Internet-related topics, such as searching a physician online. That could have led to higher awareness levels.

## Multimedia Appendix 1

The 28-item questionnaire.

## Abbreviations

SHI: statutory health insurance
PHI: private health insurance
WHO: World Health Organization
WHO-5: World Health Organization Well-being Index
