# Supplementary material for: Physician Choice Making and Characteristics Associated With Using Physician-Rating Websites: Cross-Sectional Study
Source: J Med Internet Res. 2013 Aug 28;15(8):e187. doi: 10.2196/jmir.2702 (PMC3758064; doi:10.2196/jmir.2702)
Supplement: Supplementary file 1 [file jmir_v15i8e187_app1.pdf]

Physician rating websites: a cross-sectional study assessing the impact on physician choice making and characteristics associated with its use

APPENDIX - Survey

Martin Emmert[1], Florian Meier[2], Frank Pisch[1], Uwe Sander[3]

[1] Junior Professor for Health Services Management, Institute of Management (IFM), Friedrich-Alexander-University Erlangen-Nuremberg, Germany

[2] Chair of Health Management, Institute of Management (IFM), Friedrich-Alexander-University Erlangen-Nuremberg, Germany

[3] University of Applied Sciences and Arts, Hannover, Germany

Address for correspondence:

Friedrich-Alexander-University Erlangen-Nuremberg

School of Business and Economics, Institute of Management (IFM)

Junior Professor for Health Services Management

Prof. Dr. Martin Emmert

Lange Gasse 20

90403 Nuremberg

Phone: 0911-5302253

Fax: 0911-5302114

Email: Martin.Emmert@fau.de

[1] Have you ever used the internet to find information about a physician?

Yes
No

[2] Which sources do you use when searching for a physician? [multiple response possible]

Recommendations from family, friends or colleagues
Printed business directories (e.g., yellow pages)
Information from (professional) journals and newspapers
Online-physician directories / physician rating websites
Online-search engines (e.g., Google)
Recommendations from your (general) practitioner
Physician / practice websites
Others

[3] Do you wish more transparency about the quality of care of physicians?

Yes
No

[4] What information is important for you when choosing a new physician? [multiple response possible]

Parking space
Other patients‘ opinions about this physician
Practice equipment
Scientific publications
Distance between practice and my home place
Physician’s certificates and further training
Accessibility by public transport
Physician’s specialization
Others

The next section is about your personal experiences, attitudes and opinions regarding physician rating websites. We would like to know, which sources you use when searching for a physician and whether you have used physician rating websites for this purpose.

[5] Have you ever heard of any of the following PRWs? [multiple response possible]

Sanego.de
Docinsider.de
Jameda.de
Esando.de
Onmeda.de
Imedo.de
Medführer.de
Weisse Liste (Arztnavigator)
Arzt-auskunft.de
I don´t know any of these websites.

[6] Have you ever searched for a physician on any of the following PRWs? [multiple response possible]

Medführer.de
Arzt-auskunft.de
Sanego.de
Jameda.de
Docinsider.de
Weisse Liste (Arztnavigator)
Esando.de
Onmeda.de
Imedo.de
I have not used any of these websites to search for a physician.

[7] Have you ever posted a rating on any of the following PRWs? [multiple response possible]

Imedo.de
Arzt-auskunft.de
Medführer.de
Sanego.de
Onmeda.de
Weisse Liste (Arztnavigator)
Docinsider.de
Esando.de
Jameda.de
I have never rated any physician on a physician rating website.

[8] Have you ever, based upon the publicly published results on any PRW …

	Yes	No
... selected a particular physician?		
... not selected a particular physician?		

Physician rating websites provide a variety of information about a physician. There is information about the physician himself (e.g., age, gender, languages, nationality, education) as well as about the practice in general (e.g., consultation hours, equipment, parking spaces). Additionally other patients' rating results are indicated. The illustration below shows an exemplary physician's profile from the website jameda.

[9] Of what importance is the information listed in the following for you when choosing a physician?

	No importance	Very high importance
Pictures of furnishing and equipment		
Physician's education (e.g., university)		
Gender		
Accessibility by public transport		
Technical equipment (e.g., MRT, CT, 3D-ultrasound)		
Physician's further training		

Proximity between practice and my place of residence
Consultation hours
Physician's specialization
Picture of the physician
Parking spaces
Other patients' opinions about this physician
Scientific publications
Map and directions

Some physician rating websites provide the possibility to filter results according to particular characteristics, e.g., a practice's specializations, insurances accepted or employee-friendly consultation hours. An example of such a filter is shown in the following illustration.

[10] Now we would like to assess the usefulness of different filter characteristics for your physician choice.

	Not useful	Highly useful
Filtering for very positive patient ratings		
Filtering for special consultation hours (e.g., evening or weekend)		
Filtering for accepted insurances (e.g., physician only treats privately insured patients)		
Filtering for a minimum number of ratings		
Filtering for the assessment of the waiting time to get an appointment		
Filtering for particular additional training courses or further education of a physician		
Filtering for the physician's gender		
Only show practices for which pictures are provided		
Filtering for particular specializations of the physician		
Filtering for the assessment of the waiting time within the practice		

The physician rating website jameda provides a physician's recommendation based on different categories (e.g., treatment, information, trust) which are aggregated to an overall score. Additionally, it is possible to leave a short narrative comment. The following illustration shows an exemplary rating.

[11] Please estimate each category's importance to you when choosing a physician.

	Not important	Highly important
(1) Other patients' satisfaction with the treatment		
(2) Other patients' opinions about the quality of the treatment information provided by the physician		
(3) Other patients' opinions about their relationship of trust with the physician		
(4) Assessment of the time the physician spends with the patient		
(5) Other patients' assessments of the physician's friendliness		
Overall score based on the categories mentioned above (1-5)		
Other patients' experience reports (narrative comments)		

Now, we turn towards the narrative comments. Here patients can report about their personal experiences with a particular physician without any guidelines. For example, they comment

“Competent, experienced physician, hardly any waiting times”, “Practice very well organized, hardly any waiting time, physician seems to be very competent” but also “unfriendly person, despite having an appointment, I had to wait at least one hour and that happened three times”. We would like to know how important these narrative comments are for your physician choice.

[12] Do you read available narrative comments other patients left attached to the ratings?

Yes

No

[13] What is the importance you attribute to other patients‘ narrative comments for the significance of a rating?

	No importance	Very high importance
Importance of narrative comments for a rating’s significance		

[14] What is more important to you when choosing a physician? The overall score or the narrative comments?

Overall score

Narrative comments

Equally important

[15] How useful are the following functions of a physician rating website for you?

	Not useful	Highly useful
Write a narrative comment about my physicians myself		
Read other patients‘ narrative comments about a physician		
Conduct of an online-search including all general practitioners, specialists and dentists in Germany		
Obtain information about a physician and his practice before the first visit		
Conduct of online-physician-search for a general practitioner, specialist or dentist close to my place of residence		

[16] What do you think about the overall usefulness of physician rating websites?

	Not useful	Highly useful
Benefit from the information provided on these websites		

[17] What are your major criticisms?

Criticisms

[18] Please indicate your gender.

Male

Female

I wish not to answer this question.

[19] Please indicate your year of birth. [e.g., 1984]

Year of birth

I wish not to answer this question.

[20] Please indicate your marital status.

- Married
- Single
- Divorced
- Widowed
- I wish not to answer this question.

[21] Please indicate your postal code. [e.g., 80469 for Munich]

- Postal code
- I wish not to answer this question.

[22] What is your highest level of education?

- Matura examination
- Technical university entrance qualification
- Intermediate secondary school
- Polytechnic secondary school
- Secondary general school
- Without school qualification
- I wish not to answer this question.

[23] Please indicate your current occupation.

- Self-employed
- Civil servants
- Employee
- Apprentices
- Unemployed
- Pensioners
- Students (pupils)
- Students (Univ./tech. univ.)
- Others
- I wish not to answer this question.

[24] Please indicate your current insurance status.

- Statutory health insured
- Privately health insured
- Not health insured
- I wish not to answer this question.

[25] How frequently have you seen a physician/dentist in the past six months?

- I have not received medical treatment.
- Once
- Twice
- Three times
- Four times
- Five times and more

I wish not to answer this question.

By the following two questions we would like to know how frequently you use the internet and which websites you visit mainly.

[26] How frequently do you use the internet?

Several times a day
Once per day
Several times a week
Once per week
Several times a month
Once per month
Less frequently

[27] Please indicate the purposes you used the internet for recently. [multiple response possible]

Online-shopping
Travel portals
Entertainment (e.g., music, games)
Online-communication (e.g., telephony, Email, chat)
Job offers and recruitment portals
Politics
News
Sports information
Health-related topics
Finance and insurance
Social networks
Others

[28] Please indicate for each of the five statements which is closest to how you have been feeling. Over the last two weeks…

All of the time Most of the time More than half of the time Less than half of the time Some of the time At no time

…I have felt cheerful and in good spirits
…I have felt calm and relaxed
…I have felt active and vigorous
…I woke up feeling fresh and rested
...My daily life has been filled with things that interest me
